# Child-to-Parent Violence: Attitude towards Authority, Social Reputation and School Climate

**DOI:** 10.3390/ijerph16132384

**Published:** 2019-07-05

**Authors:** Gonzalo Del Moral, Cristian Suárez-Relinque, Juan E. Callejas, Gonzalo Musitu

**Affiliations:** Department of Education and Social Psychology, Pablo de Olavide University, 41013 Seville, Spain

**Keywords:** child-to-parent violence, social reputation, attitude towards authority, school climate, adolescence

## Abstract

Research into child-to-parent violence (CPV) has focused mainly on the description of individual and family variables of adolescents. It is observed that the school context has received little attention despite being a context of development of great importance. In order to deepen the understanding in this field, the objective of this study was to analyze the relationships between child-to-parent violence (CPV) and the attitude towards authority, social reputation and school climate. A total of 2101 Spanish adolescents (50.1% males and 49.9% females) from 13 to 18 years participated. A multivariate factorial design (MANOVA, 3 × 3) was carried out using as independent variables CPV level and age. It was found that adolescents with high CPV presented lower values of positive attitude towards institutional authority (PATIA) and school climate (involvement, friendships and teacher’s help), and higher values of positive attitude towards the transgression of social norms (PATTSN) and of perceived and ideal non-conformist social reputation (PNCSR and INCSR, respectively). Younger participants obtained the highest PATIA scores and lowest of PNCSR and the 15–16 years age group obtained the highest scores in PATTSN and INCSR. Adolescents aged 17–18 years show the highest scores in involvement and teacher’s help. Also, three interaction effects were found and indicated that there is an improvement in attitudinal and school adjustment indicators according to the age, except in ideal non-conformist social reputation, which has important practical implications.

## 1. Introduction

Child-to-parent violence (hereinafter CPV) is defined as a pattern of abusive behavior (verbal, financial, physical or emotional) perpetrated towards a father or a mother by a son or daughter who is legally recognized as a minor and who usually continues living in the family home, to exert power and control over his or her parents [1,2]. In Spain, the total of reports of CPV to authorities has doubled in the last ten years and has gone from just under 2300 cases in 2007 to 4898 in 2016, according to the Public Prosecutor’s Office. Its 2017 report shows that in the period from 2013 to 2015, the proceedings against young people for any type of crime decreased by 10.02% while the cases of CPV increased by 5.13%. However, there is a hopeful decrease in the number of cases (from 4898 cases in 2015 to 4355 cases in 2016); this figure is the lowest in the last decade.

Although this problem is increasingly serious and prevalent, probably because its visibility is also greater, it has not aroused the same interest among researchers as other types of violent behavior in adolescents such as bullying or other types of intrafamily violence [3], although it is an increasingly recognized and penalized fact [4,5]. The still incipient research has focused on the characteristics of the aggressors and the victims and on the possible causes, from an individual point of view but also those related to the family context [1,6,7,8,9]. However, less attention has been paid to the school variables. In fact, in the meta-analyses regarding this topic made by Aroca, Lorenzo and Miró [10], Holt [1] and Martínez, Estévez, Jiménez and Velilla [11], it is observed that the school environment, together with the community, is the one that has received less attention despite being a context of great importance for the development of young people, where behavioral and academic difficulties can appear, but also resources for intervention [12,13].

The few published studies have focused mainly on the problems of adaptation and school performance [14], absenteeism, learning difficulties and school violence [15,16,17,18], expulsions and temporary removals from the school system [19] or less focus on the tasks, class participation and interest in learning [20,21]. Sempere et al. [18] state that the majority of adolescents with CPV problems in their study presented problems of adaptation and performance in secondary school, that non-attendance began at 11 or 12 years and that they changed schools more than once, sometimes due to behavioral problems.

Certain school variables have been significant predictors of CPV. Paulson, Coombs and Landsverk [22] state that disruptive behavior in class, school absenteeism, expulsion from the school and harassment towards teachers represent an important predictor of aggressive behavior towards parents by adolescents of both genders. Ibabe [23] found an important link between school failure and physical violence towards fathers and mothers, but not between school failure and verbal violence. In addition, school failure was correlated with low family cohesion, which in turn correlated with CPV. It is a very important fact due to the high figures of school failure of these adolescents, reaching 90% [24].

Nevertheless, other variables related to the school context, such as attitudes towards institutional authority, social reputation or school climate, have not aroused the same interest, despite the important role they seem to play in the expression of other types of violence in the adolescent stage, and specifically, in bullying and cyberbullying [25,26]. The attitude of adolescents towards school rules and towards teachers is closely related to their attitudes towards laws and other forms of institutional authority [27]. In this regard, adolescents who are less involved in violent behavior tend to show in the questionnaire a favorable attitude towards institutional authority, school and teachers [28].

The attitude towards institutional authority is closely related to social reputation, another psychosocial variable that has shown its link with violent behavior in different development contexts [9,29]. Adolescents build their identity through the image they receive about themselves from the significant people with whom they interact, mainly their parents, teachers, classmates and friends [30]. This feedback will largely determine the social self-perception of the adolescent’s reputation, that is, the perceived social reputation, built primarily from the reactions of the reference group, and the ideal reputation or, said otherwise, the reputation he or she would like to project among his or her peers [31]. There is a strong correlation between the attitude towards authority and social reputation in such a way that those adolescents who present a more negative attitude towards institutional authority perceive themselves as non-conformists and wish to project that image, based on rebellion, respect, leadership and power in the group [29].

Finally, several works highlighted the importance of the school climate, perceived as a predictor of violent behavior [32]. Thus, the quality of the interactions with the teaching staff, the support and respect of the teachers [33,34] and the social rejection of the classmates [35] seem to be very important elements to understand the psycho-emotional adjustment of adolescents.

So, one might ask whether these variables also intervene significantly in the expression of CPV. According to recent studies [36,37], there are a series of psychosocial factors common to various types of violence in the adolescent stage. There is a positive correlation between school violence among classmates, CPV and violence in adolescent couples, with a series of common factors, regarding individuals, family and school, as well as factors related to the peer group. Despite the interest reflected by these questions, there has been very little research aimed at deepening the relationship between these variables and the CPV. Therefore, the purpose of this research paper is to examine the relationships of CPV with the attitude towards authority, social reputation and school climate, according to the age of the adolescents.

## 2. Materials and Methods

### 2.1. Participants

The sample consisted of 2101 adolescents of both genders (50.1% males and 49.9% females) from 13 to 18 years old (Mean (M) = 15.07, Standard Deviation (SD) = 1.54), registered in 19 schools in Andalusia (Spain). The selection of the participants was carried out through a stratified random sampling [38]. The sampling units were: geographical area (urban or rural) and school ownership (public or private). Statistical analyses showed no significant mean differences in the dependent variables as a result of the specific location of the school and the type of school ownership.

Values lost by scales or subscales were obtained by the method of imputation by regression. This method assumes that the rows of the data matrix constitute a random sample of a normal multivariate population.

### 2.2. Procedure

A letter was sent to the selected schools explaining the objective and purpose of the study and requesting their participation. Two schools refused to participate in the research and another two centers were contacted. Subsequently, an interview was organized with the different directors of each school to explain the project in detail and provide the written informed consent document for parents and students, together with an explanatory letter about the investigation. After obtaining the corresponding permits, the questionnaires were administered by experienced and trained researchers. The battery of instruments was given to the adolescents in their usual classrooms during a regular class. Each student received a booklet with all the instruments so he or she could complete the questionnaire on his or her own.

The study fulfilled the ethical values of researching on a human scale: informed consent and right to information, protection of personal data and guarantees of confidentiality, non-discrimination, the principle of non-payment and the possibility of leaving the study in any of the phases as it is established in the Declaration of Helsinki and its subsequent updates. The Ethics Committee, from University Pablo de Olavide (Spain) approved this study.

### 2.3. Materials

CPV was captured with the Conflict Tactics Scales (CTS-2) [8,9,39,40]. The original scale consists of six items that measure two dimensions: verbal aggressions (for example, “I shout or I have shouted at my parents”) and physical (for example, “I hit or I have hit my parents with something that could hurt them”), using a Likert scale of five response options from 0—never, to 4—many times, which are answered for the father and for the mother separately. For this study, the internal consistency was 0.80 for the verbal aggression subscale and 0.71 for the physical aggression subscale.

The attitude towards authority was captured with the attitude towards institutional authority in adolescents scale [28,29,41,42]. This scale measures the attitude towards certain figures and institutions of formal authority, school rules, the teacher as a source of legitimate authority and as an impartial figure that establishes norms, socially established norms, the police and the law. It consists of nine elements with four response options (from 1—“I do not agree at all”, to 4—“I totally agree”) and is divided into two subscales: positive attitudes towards institutional authority, consisting of five elements (for example, “The police is there to make a better society for all of us”), and positive attitudes towards the transgression of social norms, composed of four items (for example, “It is normal to disobey teachers if there are no punishments”). In the present investigation, the internal consistency of both subscales was 0.76 and 0.79, respectively.

The social reputation was captured with the perceived non-conformist social reputation and ideal non-conformist social reputation dimensions of the Social Reputation Scale [29,43,44,45]. Through a total of 15 items with a range of responses from 1—never, to 4—always, the adolescent’s perception of his or her real non-conformist reputation was evaluated (for example, “Others think that I am a rebellious child”) and his/her ideal non-conformist reputation (for example, “I would like others to think I am a rebellious child”). The internal consistency for both subscales in this study was 0.83 and 0.75, respectively.

School climate was captured with the involvement dimension, friendships dimension and teacher’s help dimension of the School Climate Scale (SCS) [32,42,46]. This scale consists of 27 items that report on the social climate and existing interpersonal relationships in the classroom, with dichotomous response alternatives (true or false). The instrument measures three dimensions: involvement or the grade that the students participate in class (for example, “Students pay attention to what the teacher says”), friendships or the relationships that students maintain with their peers (for example, “Students help each other with homework”) and teacher’s help or the attitude of the teaching staff towards helping their students (for example, “The teacher shows interest in his students”). The internal consistency of these subscales obtained in the present study was 0.84, 0.79 and 0.89, respectively.

### 2.4. Data Analysis

Initially, the multivariate normality, the equality of variances and the homogeneity of the variance–covariance matrices of the study data were verified. A multivariate factorial analysis of variance MANOVA, 3 × 3 was carried out to verify the differences in the variables of the study: attitude towards authority (positive attitude towards institutional authority (PATIA), and positive attitude towards the transgression of social norms (PATTSN)); social reputation (perceived non-conformist reputation (PNCSR), and ideal non-conformist reputation (INCSR)), and school climate (involvement, friendships and teacher’s help) according to the independent variables “age” (13–14 years, 15–16 years and 17–18 years) and “CPV levels” (low, moderate and high). Univariate post hoc tests were applied with the sources of variation in which statistically significant differences were observed with the multivariate general test. Partial eta squared (η^2^_p_) was used to estimate the effect size, according to the indications of Cohen [47] for its interpretation: large ≥ 0.14, medium ≥ 0.06 and small ≥ 0.01.

The statistically significant results in the univariate tests were followed by specific tests between pairs of means by applying Bonferroni, in order to preserve the Type I error rate by study near the nominal value 0.05 [48].

## 3. Results

Table 1 shows the cross-distribution of CPV levels (low, moderate and high) according to age (13–14 years, 15–16 years and 17–18 years).

A correlation analysis was carried out with all the study variables. As shown in Table 2, all the variables were related, so they had to be taken into consideration in the subsequent analyses.

As shown in Table 3, the factorial MANOVA showed significant differences in the main effects of CPV levels, Λ = 0.91, *F*(14, 4172) = 14.24, *p* < 0.001, and age, Λ = 0.97, *F*(14, 4172) = 4.19, *p* < 0.001. A significant interaction effect was obtained, Λ = 0.98, *F*(28, 7522.60) = 1.51, *p* < 0.05.

### 3.1. Attitude towards Authority, Social Reputation, School Climate and CPV

The univariate analysis indicated that the PATIA, *F*(2, 2098) = 42.14, *p* < 0.001, was higher in adolescents with low CPV levels or showing no violence at all, and that adolescents with high CPV levels were those who show a lower PATIA. With respect to PATTSN, *F*(2, 2098) = 27.46, *p* < 0.001, the adolescents from the high CPV cluster showed a more transgressive attitude compared to adolescents with low or zero violence.

With respect to the results obtained about social reputation in PNCSR, *F*(2, 2098) = 100.18, *p* < 0.001, as in INCSR, *F*(2, 2098) = 39.47, *p* < 0.001, adolescents from the high CPV group obtained the highest values, and the low CPV group obtained the lowest values.

With regard to school climate, in involvement, *F*(2, 2098) = 12.91, *p* < 0.001, in friendships, *F*(2, 2098) = 11.88, *p* < 0.001, as well as in teacher’s help, *F*(2, 2098) = 16.28, *p* < 0.001, the highest scores were obtained by adolescents, boys and girls, from the low CPV cluster. In the case of adolescents who came from high or moderate CPV contexts, the results showed no significant differences in any of the three dimensions of school climate.

In all previous contrasts, the observed effect size was small, except in the case of non-conformist self-perception, where it was moderate (η^2^_p_ = 0.09) (see Table 4).

### 3.2. Attitude towards Authority, Social Reputation, School Climate and Age

Univariate analysis indicated (Table 5) that the PATIA, *F*(2, 2098) = 29.64, *p* < 0.001, was higher for boys and girls of 13–14 years old than for the other groups. Regarding the PATTSN, the boys and girls of 15–16 years old obtain the highest values, *F*(2, 2098) = 4.52, *p* < 0.05, without observing differences between the group of 13–14 years old and that of 17–18 years old.

Regarding the two dimensions of the non-conformist social reputation, no differences were found between the groups of 15–16 and 17–18 years old in PNCSR, although both groups obtained significantly higher scores than the adolescents of 13–14 years old, *F*(2, 2098) = 11.53, *p* < 0.001. With respect to the INCSR, the group of 15–16 years old scored higher than the adolescents of 13–14 years old, *F*(2, 2098) = 7.83, *p* < 0.001, and there was a decrease in the scores of the group of 17–18 years old, although it was not significant.

In addition, the results regarding two dimensions of school climate allow us to affirm that adolescents aged 17–18 years old showed higher scores in involvement, *F*(2, 2098) = 13.37, *p* < 0.001 and teacher’s help, *F*(2, 2098) = 4.29, *p* < 0.05, than the other two groups of adolescents, for whom the results regarding involvement were the same. No significant differences were found concerning friendships for the three age groups.

### 3.3. Interaction Effects

Three interaction effects were statistically significant in PATIA, *F*(4, 2092) = 3.69, *p* < 0.01, friendships, *F*(4, 2092) = 4.18, *p* < 0.001, and teacher’s help, *F*(4, 2092) = 2.72, *p* < 0.05 (see Table 6).

**Figure 1:**
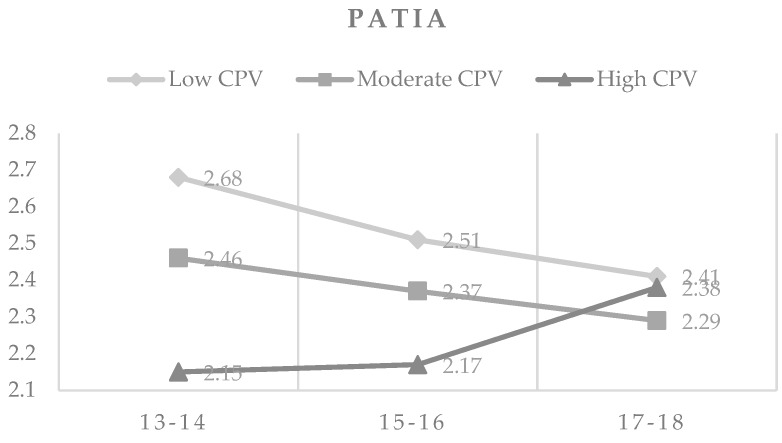
Means in PATIA by CPV levels and age.

As shown in Figure 1, PATIA tended to decrease with age, both in the low and moderate CPV levels, while in the high CPV group increased with age.

**Figure 2:**
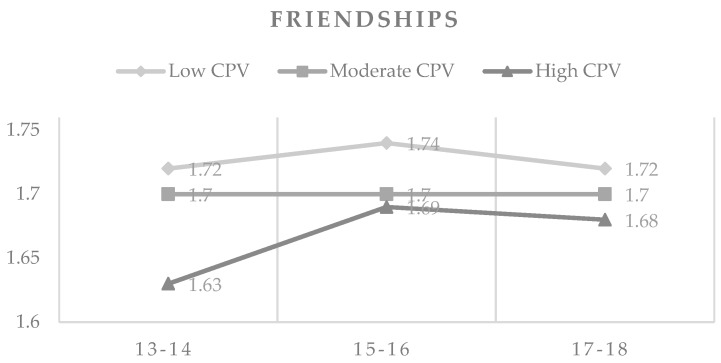
Means in friendships by CPV levels and age.

With respect to friendships, it was observed that adolescents of 15–16 years with low violence rates had higher scores than the 12–14 years age group of low CPV. Both scored higher than the group of 12–14 years of high CPV, which, on the other hand, obtained the lowest score in friendships of all groups. Regarding moderate CPV, it was observed that there were no differences in any of the age groups. There were also no differences between older adolescents (17–18 years) regardless of the level of CPV (see Figure 2).

**Figure 3:**
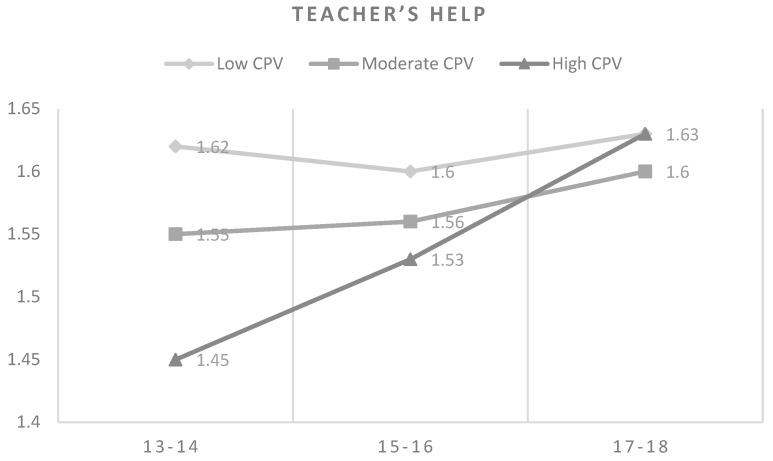
Means in teacher’s help by CPV levels and age.

Finally, the teacher’s help in the school offered the highest value for adolescents of 17–18 years with high CPV as well as for adolescents of the same age in low CPV levels. In contrast, the lowest scores of teacher’s help were observed for adolescents with high CPV of 13–14 years old, followed by the adolescents of 15–16 years old. Comparing that item by age groups, the highest values of help were obtained for adolescents of 17–18 years old in the three levels of violence. It should also be noted that with both moderate and high CPV, the teacher’s help increased according to the age. (see Figure 3).

## 4. Discussion

The aim of the investigation was to examine the relationships between the CPV and the attitude towards authority, social reputation and school climate, according to the age of the adolescents. A fundamental variable in the participation of adolescents in violent behaviors is their attitude towards authority figures and regarding the transgression of social norms [41,49]. These attitudes are learned from relationships with parents and teachers, who are the first authority figures to whom young people relate [50]. Although these attitudes may have begun to develop previously, adolescence is a fundamental stage in its consolidation, observing differences according to age.

From the results of the present study, one can observe that PATIA levels decreased as CPV levels increased, an inverse relationship to the one observed respecting to PATTSN. That is, the higher the CPV, the lower the positive attitude towards other authority figures such as teachers or the police and the higher the positive attitude to transgress the established social norms. In this sense, Moreno et al. [42] found that the negative interaction between parents and children affects other levels of social relationships of adolescents, for example, with teachers as figures of formal authority, thus increasing the levels of discontent, disenchantment and rebellion towards adult figures that impose norms that characterize this evolutionary stage [9,51].

As stated by Jaureguizar and Ibabe [52] and Fagundes et al. [53], the probability of psychological and physical aggressions against teachers increases in the cases of adolescents who assault their parents. Sancho-Acero [54] observed that 38% of the adolescents who commit aggressions against their parents have difficulties with authority figures. Because of this, the attitude towards authority could play an important mediating role in this type of school aggression. A further in depth research of this topic could be interesting, especially after the Spanish teachers’ trade union ANPE drew attention to the alarming data of 2017 in Spain. From a total of 2249 complaints received from teachers, 12% corresponded to physical aggressions and threats from adolescent students, a percentage four times higher than the one in 2016.

The conformation of these attitudes seems to follow a specific process depending on the age of the adolescents. The PATTSN reached the highest values at 15–16 years and decreased at higher ages, a figure that coincides with the up and down trajectories of forms of misbehavior, such as absenteeism, disobedience or running away from home, which are characteristics of the “adolescent-limited” route [55].

It is interesting to highlight that, despite the fact that the PATIA was higher for the group of boys and girls of 13–14 years and decreased with age, in the case of adolescents with a high CPV, the values of the PATIA rose at 17–18 years, being higher than those for adolescents of the same age with low or moderate violence rates, and being even higher than the values for almost all lower age groups with moderate or high CPV. Therefore, there is a hopeful change of attitude in late adolescence for adolescents in situations of high violence, because they are more favorably oriented towards the figures of authority. The need for someone with authority who gives structure to the environment by the means of rules and limits, which allows the adolescents to move on towards adulthood and overcome family problems, could be an important motivation related to the above-mentioned change of attitude.

The findings related to the non-conformist social reputation are very similar to the ones above. The higher the levels of CPV, the higher the scores of PNCSR and INCSR: adolescents who attack their parents perceived themselves and wanted to be perceived as challenging, hard and disobedient. This process of self-perception based on the images they receive about themselves from the significant people with whom they interact, mainly from their group of classmates and friends, is of vital importance for the behavior and relationships established among adolescents [30,31,43], and non-conformist perceptions are a risk factor for participation in violent behavior [44,45,49,50,56,57]. This finding is new and partially contradicts the concept of new CPV defended by Pereira and Bertino [58]. According to these authors, the most frequent current CPV is characterized by the presence of violence only towards parents, whereas excessively adapted behaviors in other contexts such as school can be observed, such as a conformist social reputation in school.

The PNCSR increased with age, although from 15–16 years on there were no differences found whereas the INCSR decreased from that age on. Focusing on the group of adolescents of 17–18 years, it was observed that the maintenance of high values for PNCSR, which presumably has been strengthening in previous years, contrasts with the tendency, although not significant, of the decreasing desire to be perceived by the others as a tough and challenging person. These data can be explained by the Reputation-Enhancing Goals Theory [31]: the adolescents that present a greater risk of participation in violent behaviors choose for a commitment with objectives characterized by an aggressive and non-conformist attitude, since they present a more positive valence and allow greater benefits for their status, leaving out more adjusted evolutionary goals more typical of their age [29].

But does violence towards parents increase the reputation of these adolescents? According to Castañeda et al. [36] adolescents tend to hide this type of violence, because it does not provide status in the peer group, but social sanction. Adolescents often intimidate their parents not to tell the adolescents’ friends, partner or even teachers about the climate of violence at home. In other words, despite the fact that non-conformist social reputation is related to CPV, the ideal self, regarding the relationships with parents would tend to present less non-conformist values, namely for the group of adolescents of 17–18 years old. Therefore, a reputation depending on the violence against the parents and, consequently, an INCSR in this sense, would be a risk factor for the increase in the levels of CPV aggressions.

In terms of school climate, the scores for involvement, friendships and teacher’s help were higher in contexts with low CPV. In the case of adolescents with high or moderate CPV contexts, the results did not show significant differences in any of the three dimensions. Depending on the age, the group of 17–18 years obtained the highest values of involvement and teacher’s help, whereas no differences were found in the case of friendships. It should be emphasized that adolescents aged between 17 and 18 years with high CPV obtained the highest levels of teacher’s help, followed by those of the same age group with low CPV. These levels of teacher’s help were higher than those for the rest of the age groups and violence levels. Therefore, we can see another indicator of improvement regarding the group of older adolescents, as well as an increase in the perception of the teacher’s help as the adolescents grow older, as well for moderate as for high CPV groups.

Linking this fact with the one referring to PATIA, the data suggest that at the age of 17–18 years the attitude towards authority figures others than the parents and the perception of help and support in the school context coming from adults would increase. It can be hypothesized that violent conflicts are more and more focused on and limited to intrafamilial interactions with parents, as there is a greater interest found in establishing positive and supportive relationships with other significant adult figures, such as teachers. This fact is not observed for adolescents of 13–14 years and 15–16 years, who seem to perceive the teaching staff and other adult figures in a less positive way.

Regarding the friendships, it is very interesting to note that adolescents aged 13–14 years in high CPV contexts obtain the lowest scores, which is a very interesting fact considering that levels of friendships tend to remain stable over time and do not vary according to age. This difficulty at 13–14 years, which coincides with the entry into the stage of secondary education, can be related to two elements of great interest: on the one hand, the choice of friendships and, on the other hand, the sociometric status in class. It could be that minors who attack their parents more often have to face additional difficulties in the first years of secondary school that lead them to choose peers that also present relational difficulties [59].

Furthermore, there may be the risk to be catalogued under the status label of rejected, controversial or ignored with the consequences that this entails. The experience of being more or less accepted by the peer group in the school context supposes important consequences for the psychological well-being of children and adolescents, since the experience of rejection is associated with discomfort, depressive symptomatology, feeling of loneliness, suicidal ideation and, regarding the present object of study, with the manifestation of violent behavior [49,60,61].

## 5. Conclusions

The findings of this study have important practical implications. First, there is an improvement in several attitudinal indicators and in school performance as age increases, closely related to the ability to participate properly in normative contexts and contexts regulated by adults. It has been found that the perceived environment in which adolescents are raised plays an important role in their adoption of normative and health behaviors [62]. Supporting the transition for young people of middle adolescence in the school context, even by designing specific itineraries reaching beyond the compulsory school period in Spain (16 years), could be a way to facilitate the engagement with more normalized developmental goals.

Secondly, two strategies to balance peer-pressure and authority disrespect and challenging with reducing CPV are proposed: in one hand, the inclusion of adult figures of authority in the intervention programs at school, who provide high levels of support, for example, a professional in social education, a sports mediator or a mentor. In this way, the construction of a perceived image of the adult environment in the school context in which support, acceptance and emotional involvement overcome the perception of control, coercion and norms could be enhanced.

On the other hand, the formation of school mediation teams that include adolescents with moderate and high levels of non-conformist social reputation could also be effective, focusing on the work of discovering and enhancing the benefits of positive social leadership, linked to conformist social reputation and PATIA. Work with the heroic imagination or with the construction of positive and support-based non-conformist identities (for example, identities linked to hip-hop music) seems to be giving good results in other areas such as the prevention of school bullying [63,64].

Third, it seems important to support the difficulties to establish relations of friendships that adolescents of 13–14 years old seem to have, right at the point of entry to secondary school, and that can lead to being rejected or ignored and to the association with minors with relational problems. Training in social skills as well as mentoring by older adolescents who have overcome violence problems could be two strategies to carry out in the first years of secondary school.

Fourth, it is necessary to send a message of encouragement and hope to parents, professionals and teachers, since certain indicators seem to improve according to age, showing a clear improvement at 17–18 years old. The figure of “mediators and assistants” parents can be a resource that allows families suffering CPV to receive support and advice in the school context, especially during the first years of secondary school.

This last point, however, is related to one of the main limitations of the study: the transversal nature of the study impedes establishing a causal relationship between the studied variables, so it would be necessary to carry out longitudinal studies to deepen the findings. In fact, the improvement in the group of 17–18 years could be due to the fact that a considerable number of adolescents drop out of secondary school at the age of 16, while those with a greater number of protective factors remain studying.

Future research would study the role that sex could have in relation to the variables studied. It is known that boys who assault their parents are more likely to use violence in extrafamilial contexts with respect to girls [2,15], so they would be likely to also present higher values of non-conformist social reputation and PATTSN.

## Figures and Tables

**Table 1:** Distribution of the sample according to age and child-to-parent violence (CPV) levels.

Age		CPV			Total
		Low	Moderate	High	
13–14	*N*	594	209	43	846
	%	70.2	24.7	5.1	100
15–16	*N*	483	259	66	808
	%	59.8	32.1	8.2	100
17–18	*N*	262	155	30	447
	%	58.6	34.7	6.7	100
Total	*N*	1339	623	139	2101
	%	63.7	29.7	6.6	100

**Table 2:** Correlations between attitude towards authority, social reputation and school climate on the one hand and CPV on the other hand.

	1	2	3	4	5	6	7	8	9	10	11
1. PATIA	1										
2. PATTSN	−0.172 **	1									
3. PNCSR	−0.305 **	0.370 **	1								
4. INCSR	−0.220 **	0.306 **	0.593 **	1							
5. Involvement	0.215 **	−0.177 **	−0.117 **	−0.073 **	1						
6. Friendships	0.172 **	−0.153 **	−0.109 **	−0.105 **	0.297 **	1					
7. Teacher’s help	0.372 **	−0.179 **	−0.176 **	−0.124 **	0.323 **	0.283 **	1				
8. Physical violence Mother	−0.091 **	0.120 **	0.196 **	0.131 **	−0.045 *	−0.074 **	−0.052 *	1			
9. Verbal violence Mother	−0.236 **	0.184 **	0.367 **	0.244 **	−0.119 **	−0.092 **	−0.116 **	0.322 **	1		
10. Physical violence Father	−0.089 **	0.073 **	0.160 **	0.056 *	−0.029	−0.020	−0.020	0.455 **	0.189 **	1	
11. Verbal violence Father	−0.202 **	0.131 **	0.271 **	0.158 **	−0.076 **	−0.080 **	−0.080 **	0.193 **	0.717 **	0.308 **	1
M (SD)	2.49 (0.57)	1.61 (0.59)	1.39 (0.41)	1.21 (0.29)	1.45 (0.20)	1.72 (0.17)	1.59 (0.22)	0.04 (0.19)	0.94 (0.75)	0.03 (0.16)	0.71 (0.66)

Note: PATIA: positive attitude towards institutional authority; PATTSN: positive attitude towards the transgression of social norms; PNCSR: perceived non-conformist reputation; INCSR: ideal non-conformist reputation; ** *p* < 0.01; * *p* < 0.05.

**Table 3:** Multivariate (M)ANOVA (3^a^ × 3^b^) for the different variables under study.

Source of Variation	Λ	*F*	*DF*_between_	*DF*_error_	*p*	η^2^_p_
(A) CPV levels ^a^	0.91	14.24	14	4172	<0.001 ***	0.05
(B) Age groups ^b^	0.97	4.19	14	4172	<0.001 ***	0.01
A × B	0.98	1.51	28	7522.60	<0.05 *	0.00

Note: a_1_, Low CPV, a_2_, Moderate CPV, a_3_, High CPV; b_1_, 13–14 years, b_2_, 15–16 years, b_3_, 17–18 years. *** *p* < 0.001; * *p* < 0.05.

**Table 4:** Means, standard deviations, *F* values and effect size between CPV levels and the variables under study.

	CPV			*F*(2, 2098)	η^2^_p_
	Low	Moderate	High		
*Attitude towards authority*					
PATIA	2.57 (0.57) ^a^	2.38 (0.54) ^b^	2.21 (0.53) ^c^	42.14 ***	0.04
PATTSN	1.54 (0.56) ^c^	1.69 (0.62) ^b^	1.85 (0.66) ^a^	27.46 ***	0.03
*Social reputation*					
PNCSR	1.31 (0.34) ^c^	1.48 (0.42) ^b^	1.74 (0.63) ^a^	100.18 ***	0.09
INCSR	1.18 (0.25) ^c^	1.25 (0.32) ^b^	1.38 (0.43) ^a^	39.47 ***	0.04
*School climate*					
Involvement	1.47 (0.21) ^a^	1.43 (0.20) ^b^	1.41 (0.20) ^b^	12.91 ***	0.01
Friendships	1.73 (0.17) ^a^	1.70 (0.18) ^b^	1.67 (0.17) ^b^	11.88 ***	0.01
Teacher’s help	1.61 (0.22) ^a^	1.57 (0.22) ^b^	1.53 (0.23) ^b^	16.28 ***	0.02

Note: *** *p* < 0.001; a > b > c.

**Table 5:** Means, standard deviations, *F* values and effect size between age groups and the variables under study.

	Age			*F*(2, 2098)	η^2^_p_
	13–14	15–16	17–18		
*Attitude towards authority*					
PATIA	2.60 (0.61) ^a^	2.44 (0.54) ^b^	2.37 (0.51) ^b^	29.64 ***	0.03
PATTSN	1.59 (0.59)	1.65 (0.62) ^a^	1.56 (0.55) ^b^	4.52 *	0.00
*Social reputation*					
PNCSR	1.34 (0.35) ^b^	1.42 (0.44) ^a^	1.43 (0.44) ^a^	11.53 ***	0.01
INCSR	1.19 (0.27) ^b^	1.24 (0.32) ^a^	1.21 (0.28)	7.83 ***	0.01
*School climate*					
Involvement	1.44 (0.19) ^b^	1.45 (0.21) ^b^	1.50 (0.21) ^a^	13.37 ***	0.01
Friendships	1.72 (0.16)	1.73 (0.17)	1.71 (0.19)	1.20 n.s.	0.00
Teacher’s help	1.59 (0.22)	1.58 (0.22) ^b^	1.62 (0.22) ^a^	4.29 *	0.00

Note: *** *p* < 0.001; * *p* < 0.05; n.s. = non significant; a > b.

**Table 6:** Means, standard deviations and post-hoc comparisons between age groups and CPV levels for positive attitude towards institutional authority (PATIA), friendships and the teacher’s help.

	Age	CPV			*F*(4, 2092)	η^2^_p_	Post-Hoc
		Low	Moderate	High			
PATIA	13–14	2.68 (0.61) ^a^	2.46 (0.56) ^b2^	2.15 (0.63) ^c^			a > b_1,_ b_2,_ b_3_ > c; a > b_2_ > c; a > b_3_ > c
	15–16	2.51 (0.53) ^b1^	2.37 (0.54) ^c^	2.17 (0.44) ^c^	3.69 **	0.01	
	17–18	2.41 (0.50) ^b3^	2.29 (0.50) ^c^	2.38 (0.56) ^c^			
Friendships	13–14	1.72 (0.17) ^a2^	1.70 (0.16)	1.63 (0.17) ^b^			a_1_ > a_2_ > b
	15–16	1.74 (0.17) ^a1^	1.70 (0.17)	1.69 (0.17)	4.18 ***	0.01	
	17–18	1.72 (0.18)	1.70 (0.20)	1.68 (0.15)			
Teacher’s help	13–14	1.62 (0.21) ^a3^	1.55 (0.23) ^b2^	1.45 (0.24) ^b4^			a_1_, a_4_, a_5_ > b_4_; a_2_, a_3_ > b_1_, b_2_, b_4_; a_2_ > b_3_
	15–16	1.60 (0.22) ^a5^	1.56 (0.21) ^b1^	1.53 (0.23) ^b3^	2.72 *	0.01	
	17–18	1.63 (0.22) ^a2^	1.60 (0.23) ^a4^	1.63 (0.20) ^a1^			

Note: *** *p* < 0.001; ** *p* < 0.01; * *p* < 0.05.
